# Optical Multimode Fiber-Based Pipe Leakage Sensor Using Speckle Pattern Analysis

**DOI:** 10.3390/s23208634

**Published:** 2023-10-22

**Authors:** Jonathan Philosof, Yevgeny Beiderman, Sergey Agdarov, Yafim Beiderman, Zeev Zalevsky

**Affiliations:** 1 The Nanotechnology Center, Faculty of Engineering, Bar-Ilan University, Ramat-Gan 5290002, Israel; jonathan.philosof@gmail.com (J.P.);; 2 Holon Institute of Technology (HIT), Faculty of Electrical Engineering, Holon 5810201, Israel

**Keywords:** speckle pattern, optic fiber sensor, machine learning (ML), leak detection

## Abstract

Water is an invaluable resource quickly becoming scarce in many parts of the world. Therefore, the importance of efficiency in water supply and distribution has greatly increased. Some of the main tools for limiting losses in supply and distribution networks are leakage sensors that enable real-time monitoring. With fiber optics recently becoming a commodity, along with the sound advances in computing power and its miniaturization, multipurpose sensors relying on these technologies have gradually become common. In this study, we explore the development and testing of a multimode optic-fiber-based pipe monitoring and leakage detector based on statistical and machine learning analyses of speckle patterns captured from the fiber’s outlet by a defocused camera. The sensor was placed inside or over a PVC pipe with covered and exposed core configurations, while 2 to 8 mm diameter pipe leaks were simulated under varied water flow and pressure. We found an overall leak size determination accuracy of 75.8% for a 400 µm covered fiber and of 68.3% for a 400 µm exposed fiber and demonstrated that our sensor detected pipe bursts, outside interventions, and shocks. This result was consistent for the sensors fixed inside and outside the pipe with both covered and exposed fibers.

## 1. Introduction

The major problems for potable water supply worldwide are water availability and quality and losses in the supply mains and distribution networks. Numerous studies indicate water losses of 20–30%, while in developing countries, where the rarely maintained pipes often serve for more than half a century, the losses could reach 50% [1,2]. According to the World Bank, an amount of water worth $15 billion is lost worldwide each year. In Asia alone, 29 billion cubic meters of water worth $2 billion are lost annually [3]. In addition to the typical system losses, developing areas are often affected by illegal connections, further reducing the efficiency and profitability of the water supply. This research focuses on the development and testing of a laser-based multimode optic fiber sensor equipped with machine learning (ML) software that allows for water supply monitoring, including leak detection and quantification, evaluation of transient flows, and alerts for any illegal interventions or sabotage.

A few types of sensors are viable for the detection of water pipe leakage, and they range from very simple to complex and expensive. These sensors can be divided into active and passive types. Active sensors are typically used to follow up on a known problem or for maintenance. They use a probe signal to evaluate the state of the system and detect anomalies. Conversely, passive sensors monitor continuously. They do not add energy to the system and rely on leaks to provide detectable fluctuations. Our research focuses on a passive sensor [4,5,6].

Detecting leaks in water pipelines is a challenging task, as a small leak may not produce noticeable changes in the normal operation of the system. Moreover, the background noise in water transmission systems can be relatively high, resulting in a low signal-to-noise ratio (SNR). Several leakage detectors are currently in use. Noise loggers sense the sound propagating along a pipe and typically use a piezoelectric microphone fixed on a pipe or valve. They are expensive to deploy, as the systems require the installation of numerous sensors. The sensor detects a leak by comparing the recorded sound with a known baseline using sophisticated filtering and detection algorithms. The system is most effective under reduced noise, which is most commonly during nighttime, when water consumption is minimal. For PVC pipes, the sensors need a longer time to gather data and need to be closer together [1,7,8]. A smart meter can be composed of a water meter (usually a flow meter) transmitting data to a centralized hub. While this is not a direct method for leak detection, the data gathered can help to detect a leak. A smart meter is configured to flag water flow that never reaches zero, and it is most effective at night, when the flow is minimal. A pressure meter could also facilitate leak detection, alerting about any abnormal pressure changes [7].

Pipe leaks can also be detected with the following procedures [1,7,8,9]:

- Listening sticks are placed above the pipes, allowing an operator to hear leak-related sounds.
- Thermal imaging indicates a leak according to a difference in temperature around the pipe.
- Tethers or robotic crawlers can be used with a sensor at their end.
- Satellite-based ground-penetrating radar can detect the chemical makeup of a large portion of the soil around a pipeline.

Optic fiber sensors are being increasingly developed and applied, offering several benefits, such as durability, corrosion resistance, multiplexing capabilities, light weight, compactness, and their long-range deployment potential [10]. Several main mechanisms are currently in use for optic-fiber-based leak detection by utilizing an optic fiber as an acoustic or thermal sensor. Most of these sensors measure the light intensity variation or the wavelength shift caused by the fiber bending. Existing acoustic detectors consist of a fiber attached to or embedded in a concrete pipe. The fiber’s transmission efficiency changes with its bending radius under vibration. By monitoring the intensity and frequency of the light traveling through the fiber, a leak or fracture can be identified. These sensors are usually sensitive to higher modes and are prone to fiber breakage due to macro-bending [11]. For a thermal-based leak detector, a fiber is used to measure the soil temperature around a pipe. Using a fiber Bragg grating (FBG), changes in the wavelength due to temperature variation are measured, and rapid temperature changes can indicate a leak. These methods require the installation of an optic fiber together with a pipe, however they cannot indicate a leak’s location, with only acoustic based sensors being able to detect concrete pipe fractures. The sensors are also relatively complex, with some requiring temperature control, sophisticated detection methods, non-conventional fibers, and more [7,9,11,12,13]. Optic-fiber-based sensors are separately used to detect pipeline damage by utilizing a variety of methods, such as distributed acoustic sensing, fiber Bragg gratings, and specklegram monitoring. These methods have increasingly adopted machine (ML) and deep learning (DL) techniques to enhance their accuracy over the conventional statistical approaches [14,15,16]. We aim to design and test a multimode optic fiber (MMF)-based sensor able to detect and quantify pipe leakage using ML to analyze the laser based speckle patterns emitted from the fiber outlet and captured by a high-speed defocused camera, thus increasing the sensor’s sensitivity. The novelty of our sensor relates to its ability to perform multiple functions, such as detecting pipe bursts, damage, transient flow, and, notably, the leak flow rate. As we additionally explore an exposed fiber version, future implementations may also be used to evaluate water quality. The sensor can use low-power lasers, be fitted to various types of existing pipes, and will enable leak and pipe burst localization in future work. We are currently investigating such possibility. The sensor’s versatility and simplicity make it attractive as an automated, comprehensive, and continuous monitoring system. Furthermore, the fiber along the pipe can simultaneously serve as a data transmission medium.

## 2. Theoretical Background

### 2.1. Speckle Pattern Analysis and Its Applications

A multimode fiber is an optical fiber with a wide enough core to accommodate multiple modes of light for a given wavelength. Laser-based coherent light passing through a MMF generates a speckle pattern at the fiber’s exit through a process of random reflections and interference between different modes of light propagating through the fiber. Deformation of the fiber can alter its reflective properties differently depending on the mode index, causing a speckle pattern variation. The fiber far-field light speckle distribution ${(A}_{0})$ is the superposition of all of the modes’ amplitudes [17,18,19,20,21,22].

(1) $$A_{0}\left(x,y\right)=\sum_{m=0}^{M}a_{0m}(x,y)e^{j\varphi_{0m}(x,y)}$$

where *M* is the number of light modes inside the fiber, and $a_{0m}\left(x,y\right)$ and $\varphi_{0m}(x,y)$ are the amplitude and phase of mode *m* of pixel $\left(x,y\right)$, respectively.

The far-field speckle pattern intensity $I\left(x,y\right)$ captured by a defocused camera from the edge of the fiber is described as:

(2) $$I\left(x,y\right)={\left|A_{0}\left(x,y\right)\right|}^{2}=\sum_{m=0}^{M}\sum_{n=0}^{M}a_{0m}\left(x,y\right)a_{0n}\left(x,y\right)e^{j{(\varphi }_{0m}\left(x,y\right)-\varphi_{0n}(x,y))}$$

where *M* is the number of light modes inside the fiber, $a_{0m}\left(x,y\right)$ and $\varphi_{0m}(x,y)$ are the amplitude and phase of mode m of pixel $\left(x,y\right)$, respectively, and $a_{0n}\left(x,y\right)$ and $\varphi_{0n}(x,y)$ are the amplitude and phase of mode n of pixel $\left(x,y\right)$, respectively [22,23].

When portions of coating and cladding are locally removed, the propagating light partially leaks from the fiber and interacts with the environment, affecting the speckle pattern at the fiber’s exit. A speckle pattern is very sensitive to tiny vibrations; a small fiber deformation can significantly affect the resulting scattering of the light and the speckle pattern at the fiber’s outlet [18,22,24]. Due to the high sensitivity, speckle pattern analysis is useful for the detection of micro-vibrations. A speckle pattern is observed when a digital camera captures a laser beam exiting the fiber’s outlet. Cross-correlation of the subsequently recorded video frames containing the speckle patterns can be used to obtain a displacement graph reflecting the fiber vibrations, as well as the light and water flow interactions in the case of an exposed fiber [22,23]. The use of speckle pattern analysis can be further improved with a defocused imaging camera, thus increasing amplitude sensitivity [25].

We intend to develop and test an MMF-based optical sensor for the detection and quantification of pipe leakage. A MMF inserted into a pipe will be affected by the liquid flow, and the defocused speckle patterns will undergo variation. We assume that the processing of the recorded video may allow continuous leak monitoring or pipe burst detection.

### 2.2. Water Pipes and Flow Characteristics

A pipe flow can be subdivided into two principal categories: laminar and turbulent, with the Reynolds number (Re) as the determining criterion. When Re exceeds 2500, the flow becomes turbulent, and the pipe head loss is increased. In the area of a leak, the flow undergoes significant local turbulence. The pipe flow is non-uniform in most real-world applications due to the variations in conduit flow systems. In the center of a pipe, the flow velocity is at a maximum, but near a pipe’s wall, it is close to zero [26,27,28]. When a pump starts, stops or a valve is suddenly closed, the flow becomes transient, creating a water hammer effect. A water hammer is a phenomenon in conduit flow systems in which a pressure surge forms due to flow variation. The forming transient-flow shockwave can cause several problems, including the rupture or collapse of a pipe [29]. Using Jukowsky’s equation, the water-hammer-related pressure variation (∆P) is expressed as:

(3) ∆P=±ρa∆V

where ∆P is the pipe pressure variation, ρ is the fluid density, a is the acoustic wave speed, and ∆*V* is the cross-sectional average water velocity variation [29].

For a steel pipe, a = 1200 [m/s], and for an HDPE pipe, a = 200–400 [m/s]. Therefore, a rapid change in the water velocity by 1 m/s causes the pressure to rise by 12 bar in a steel pipe and by around 2–4 bar in a plastic pipe.

We expect that our fiber-based sensor can detect the transient flow related to pump starting and stopping, as well as the water hammer effect.

### 2.3. Pipe Leak Hydraulics

Pipe leaks are mostly associated with high pressure and its temporal variation in a system. Lower piezometric points (resulting in high static pressure) have a higher probability of a pipe burst. In combination with the occurrence of a water hammer, which induces a short pressure surge, a critical pressure can be reached. Additional factors of an eventual pipe burst are material corrosion, pipe deformation under heavy external pressure or surge collapse, and illegal connections [30]. A simple hydraulic model of a pipe leak is described as flow through an orifice under particular pressure:

(4) $$Q=C_{d}A\sqrt{2gh}$$

where *Q* is the leak flow, *C_d_* is the discharge coefficient, *A* is the area of the leak, *h* is the pressure head, and *g* is the acceleration due to gravity [30].

We assume that the turbulent flow occurring in the leak area affects the MMF sensor, allowing the detection of a leak using ML methods for data analysis. 

### 2.4. Methods of ML Analysis

The traditional methods of speckle pattern analyses include cross-correlation of the recorded video frames or contrast imaging. When transformed into a displacement graph, the captured video can be further analyzed in the frequency domain, as well as with statistical analyses of the signal amplitude variation. In recent years, machine and deep learning applications for optical sensors have grown in prominence, particularly in the enhancement of monitoring and detection accuracy. ML algorithms process sample data—referred to as training data—to make predictions on new unclassified data without being explicitly programmed to achieve the intended goal. Classification algorithms are efficient at placing boundaries between different classes by using features that show significant separation [31]. After preliminary testing of different ML algorithms, we selected and applied the Support Vector Machine (SVM) and K-Nearest Neighbor (KNN) algorithms. These two algorithms are very adaptable for different types of data and showed at least twice the accuracy and reliability in leak detection compared to the others tested, including Naïve Bayes and neural networks.

The SVM algorithm uses a plot (linear, polynomial, etc.) to divide the space containing data points using either a hard or a soft margin. The algorithm maximizes the space between each divided region to find the best model. Subsequent data points can be classified according to the region into which they fall. Each separate region represents a specific group or event [32]. The KNN algorithm compares the number of neighbors of varying types in each data point’s region. It uses only the K closest points for this classification, grouping the closest data points together [33].

## 3. Materials and Methods

### 3.1. Hydraulic Setup and Optical System

**Figure 1:**
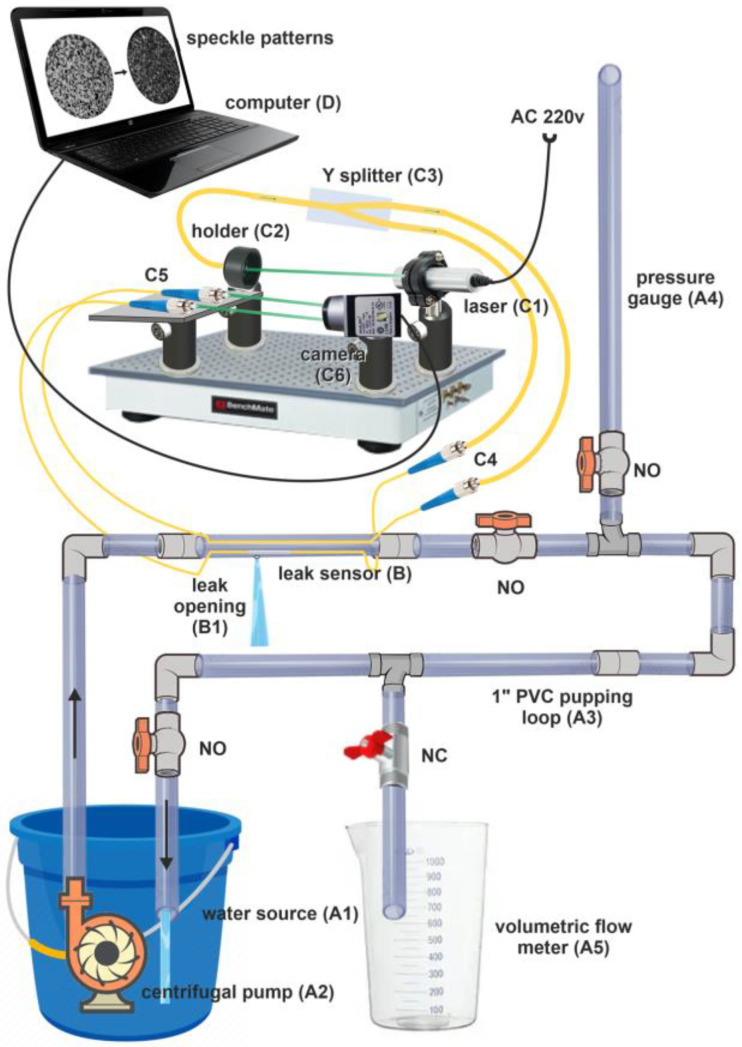
The hydraulic and optical system setup diagram. A1—water source, A2—centrifugal pump, A3—piping loop, A4—pressure gage, A5—volumetric flow meter, B—leak sensor, B1—leak opening, C1—laser, C2—holder for the fiber’s inlet, C3—fiber splitter (50/50), C4—connection to the sensor fibers, C5—stand for the fiber’s outlet, C6—digital camera, and D—computer.

To test the ability and complexity of the proposed sensing method, we constructed an optical and hydraulic setup to accommodate different configurations of our MMF-based sensor (Figure 1).

A 15 L bucket filled with water was used as a water source. It contained a centrifugal pump (SEA STAR Q-3500) with a nominal capacity of 3000 L/h under 3 m head. The pump was connected to a 1″ piping loop containing the leak sensor and equipped with valves and fittings to enable measurement of the pump flow and head, as well as the leak flow. Depending on the hydraulic system status, the loop valves could be normally open (NO) or closed (NC). The pressure gage comprised a transparent vertical branch with a valve to measure the pipe pressure in cm of the water column. The volumetric-type flow meter contained a pipe branch with an exit to a measuring burette. The optical setup contained a green laser PPGL-2100F emitting a 300 µW, 532 nm beam directed to the fiber inlet, a holder for the fiber’s inlet (limiting stray light), a fiber splitter (50/50) for recording speckle patterns from two fibers simultaneously, a connection with the sensor fibers, a stand for the fiber’s outlets, and a high-speed defocused digital camera (Basler acA1440-220 µm) set to record at 100 FPS. The camera was connected to a computer to record and process the videos containing speckle patterns.

### 3.2. The Sensor

To determine the optimal configuration of the sensor, we tested several MMF types, as well as their positioning and core exposure. Multimode fibers with core sizes of 200, 400, and 600 µm were fixed inside or outside the pipe. Some of the fibers placed inside the pipe had a portion (approximately 1 cm) of their core exposed (cladding and plastic protection were manually removed). The detection segment of the sensor was constructed from a PVC pipe (1″ diameter, 20 cm long), with the fibers running over or inside it. To fix the MMF outside the pipe, the fiber was laid over, and the pipe was covered with a plastic folio. For the internal installation of the fiber, the pipe thread was axially cut from both ends to allow the fiber’s ingress and sealed with hot glue. The fiber was fixed 5 mm from the pipe wall going over the simulated leak hole. To simulate pipe leakage, we drilled 3–8 mm diameter holes in the sensor’s pipe and plugged them until a burst or leak was simulated. The properties of the fibers are presented in Table 1.

**Figure 2:**
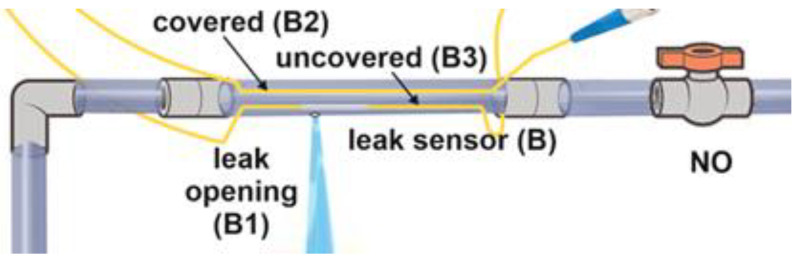
Schematic of the sensor with covered and exposed MMF.

We used the following covered and exposed fibers (covered meaning with an unexposed core): 200 µm, covered, fixed on the pipe’s surface. In addition 200, 400, and 600 µm fibers were placed inside the pipe as covered and exposed variants. An illustration of the sensor variants is shown in Figure 2 for interior installations.

Following the local removal of the fiber’s cladding, the core’s surface remained rough, and it did not allow total internal reflection. We assumed that a portion of the light leaking from an exposed fiber would exit the core to the water and would interact with its surroundings. The light returning to the core due to reflection and refraction (from the flowing water and its impurities) might carry information on the state of the water in the pipe, which would add to the abilities of the sensor.

The installation of an optic-fiber-based sensor is critical for its performance; two methods of installation were considered for field applications. The MMF could be placed above the pipe and fixed with a folio used for the protection of newly installed buried pipes. Alternatively, after pipe installation, an ordinary inspection robot could provide free fiber laying inside the pipeline.

### 3.3. Speckle Pattern Recording and Processing Software

We recorded video files containing the defocused speckle patterns captured from the fiber’s outlet. After processing the files with a MATLAB script, the amplitude of the variance between frames over time was extracted. First, the cross-correlation of the speckle pattern images was used to determine its displacement across the x and y axes of the image, given that the two images differed only by an unknown shift. When the images matched, the value of the cross-correlation function was maximized. The cross-correlation was used to analyze the positional shifting between two adjacent speckle patterns to determine each pixel’s temporal correlation [22,23].

Two speckle patterns I and I’ are usually divided into a set of sub-images. The cross-correlation algorithm tracks the movement of several speckles acting together as a sub-image. The correlation function is calculated for each pair of corresponding sub-images, and the respective displacement is derived from the maximum position difference. Sub-image A in image I is allowed to sweep over image I’. When the area with the highest statistical agreement (cross-correlation) is found, it is labeled as sub-image A’ and is considered to correspond to sub-image A. The discrete cross-correlation between A and A’ is calculated as follows:

(5) $$R_{AA^{l}}\left(d_{x},d_{y}\right)=\frac{1}{NM}\sum_{i=0}^{N-1}\sum_{j=0}^{M-1}A(i,j)A^{l}(i+d_{x},j+d_{y})$$

where *dx* and *dy* are displacements in the *x* and *y* directions, respectively. The movement over the surface position A to A’ is found according to the position of the correlation peak and is given by the displacement vector of the midpoint of sub-image A [23].

These calculations are performed for all sub-images of I until a displacement field of the whole surface is obtained, meaning that the movement is determined in two directions: x and y. The height of the correlation peak indicates how similar the cross-correlated sub-images are and, hence, yields a value of the accuracy of the measurement.

**Figure 3:**
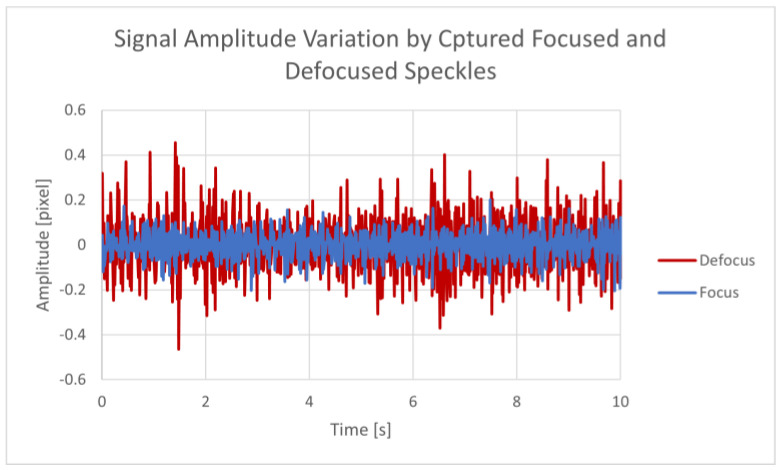
Focused vs. defocused specklegram’s signal amplitude.

The graph in Figure 3 shows the temporal changes in the correlation peak’s location in pixel units for a single recording. We extracted the position of the correlation peak and plotted its time-varying position, with the amplitude denoting the shift in the position of the correlation peak in pixel units of the camera. All relative movements were cumulatively summarized to extract the total movement vector.

Using this method, a video including 10,000 frames at a resolution of 128 × 128 pixels took about 2.5 s to process on a laptop (Intel 6700HQ).

**ML Algorithms’ Data Processing Path**

**Figure 4:**
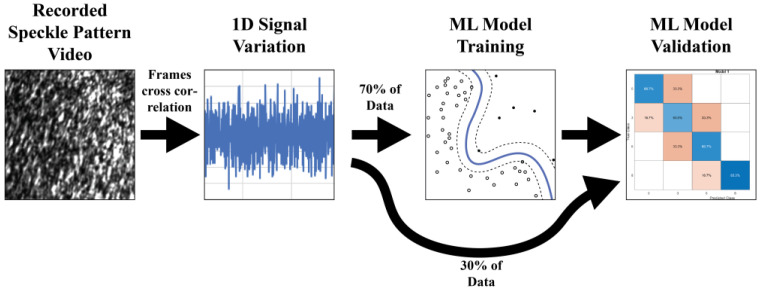
ML process diagram.

We used several supervised ML algorithms, including Naïve Bayes and neural networks, to classify the leak size and flow rate. The SVM and KNN algorithms produced the best results compared to the others tested. The data path used to create a leak detection model is presented in Figure 4. 

## 4. Results

### 4.1. Defocused Speckle Pattern

To exhibit the advantages of our defocused capture method, we tested the sensor with focused and defocused lenses. For this test, the speckle patterns from a 400 µm covered fiber were captured with an adjustable 12–14 mm lens under a water flow of 6.6 L/min and a leak diameter of 6 mm. The recordings were 10 s long, with five repetitions per state. After processing the recordings using the cross-correlation algorithm, the absolute average amplitude was calculated. Overall, the defocused speckle patterns produced an average amplitude twice that obtained from the traditional focused images. Therefore, we obtained a higher sensitivity of the sensor. A comparison between two of the tests is presented in Figure 3.

### 4.2. Pipe Burst Simulation

**Figure 5:**
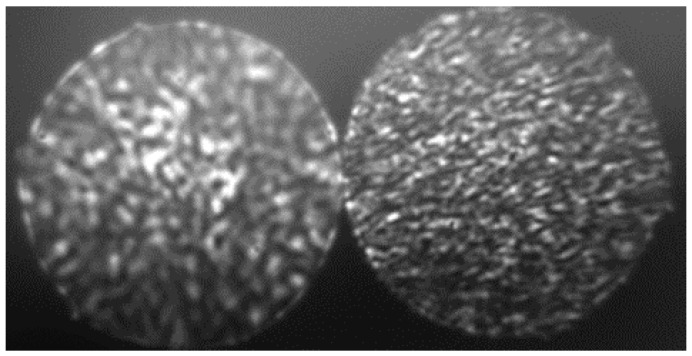
Full speckle pattern image from two simultaneously recorded MMFs—400 µm placed inside (**left**) and 200 µm placed outside (**right**).

Three sensor types were tested under multiple flow rates, and each test was repeated three times. A high-speed camera (Basler acA1440-220 µm) recorded speckle pattern videos at 100 fps with a resolution of 992 × 512 pixels. We used a 400 µm fiber placed inside a pipe and a 200 µm fiber on its outer surface (both covered), as well as two 400 µm fibers inside a pipe, with one covered and the other exposed. A green 532 nm laser illuminated the fiber inlet. The propagating light was divided 50/50 through a splitter, and the speckle patterns from each fiber’s outlet were simultaneously recorded. The speckle pattern image from the two fibers captured in the footage is presented in Figure 5.

**Figure 6:**
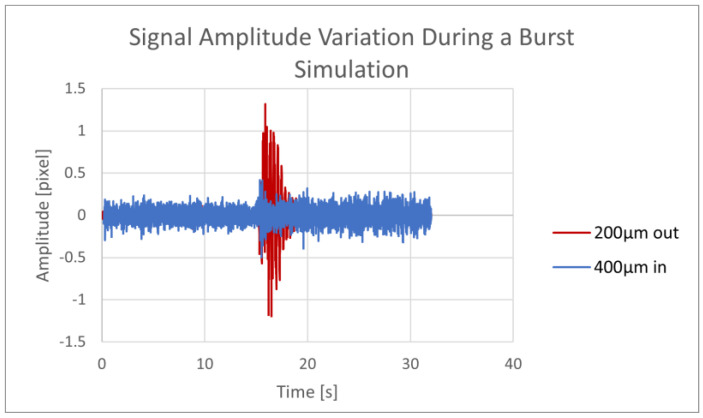
The outer and inner fibers’ signals in a pipe burst simulation (200 and 400 µm).

A pipe burst test was simulated after 15 s of unimpeded flow. A leak was instigated by the rapid removal of a pipe wall plug. The recording continued for 17 more seconds to record the steady state of the leaking system. The speckle pattern processing for one of the recording samples is given in Figure 6.

The amplitude of the signal momentarily increased as the plug was removed, showing that a strong pipe disruption, which simulated an outside intervention, affected the recorded signal. The test was repeated two more times, showing variance in the maximum signal amplitude measured with the 200 µm fiber (resulting from inaccuracy in the manually simulated “bursts”). 

With the same protocol, we repeated the burst tests using the 400 µm covered and exposed MMF sensors inserted into the pipe. Both sensors showed similar results; during the burst simulation, the maximum signal amplitude increased by up to 4.67 times. The results of the two tests comparing the maximum signal amplitudes between the undisturbed flow and burst simulation are presented in Table 2.

### 4.3. Analysis Using a Frequency Spectrogram

**Figure 7:**
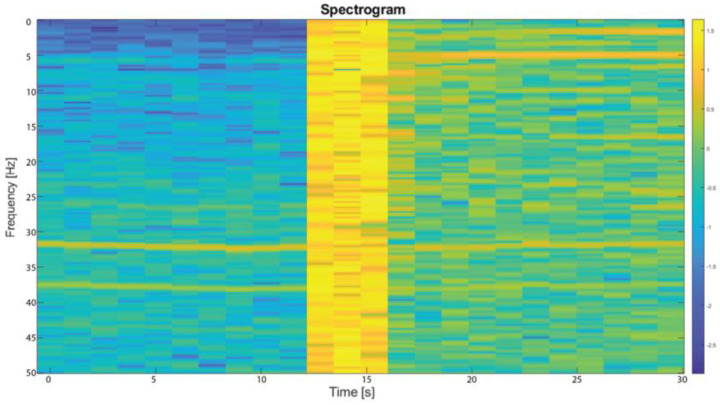
A spectrogram of a pipe burst test—400 µm covered fiber.

Another method applied for the detection of pipe bursts and leaks utilizes a frequency spectrogram. A spectrogram visualizes the variation in the frequency spectrum over time, revealing both narrow and spectrum-wide changes. The spectrogram of the speckle cross-correlation signal for a burst and further leak simulation obtained with a Fourier transformation is presented in Figure 7. An increased power for a certain frequency corresponds to a higher point on the color scale.

A clear spectrum change could be observed as the simulated burst occurred, allowing its detection. Similarly, the change in the overall frequency spectrum before and after the burst was also visible. This showed that the leak affected the sensor in the frequency domain, demonstrating another way of processing the data for leak detection.

### 4.4. Statistical Analysis

Unlike the previous tests, only one fiber’s speckle patterns were recorded, and a selected window of 128 × 128 pixels was used. The recordings were taken at a rate of 100 fps for 10 s, with three repetitions per test. For every fiber type (200, 400, 600 µm, covered or exposed, all installed inside a pipe), three leak sizes of 3, 6, and 8 mm in diameter were tested under the pipe flow rates of 2.2, 4.2, 5.3, 6, and 6.6 L/min. For each flow rate, the leak volume and velocity were evaluated. The environmental temperature was stable and relatively consistent between the tests.

**Figure 8:**
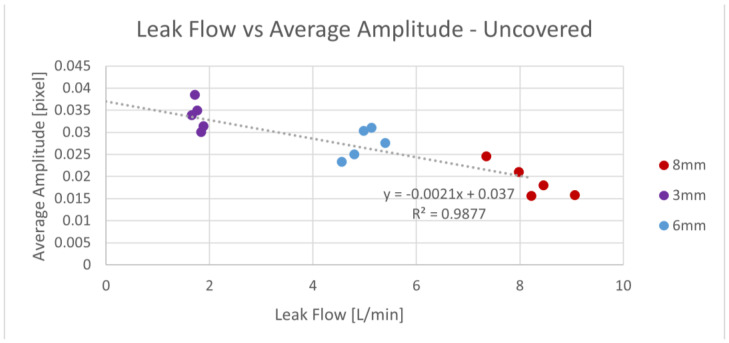
Average signal amplitude vs. leak flow for three leak diameters—400 µm exposed fiber.

We did not observe a correlation between the pipe flow and the average signal amplitude. This indicated that the pipe flow did not significantly affect the leakage discharge. However, we found a strong correlation between the average signal amplitude and the leak flow, showing the possibility of leak size detection with our sensor. The relation of the average signal amplitude and the leak flow for the 400 µm fiber is presented in Figure 8.

**Figure 9:**
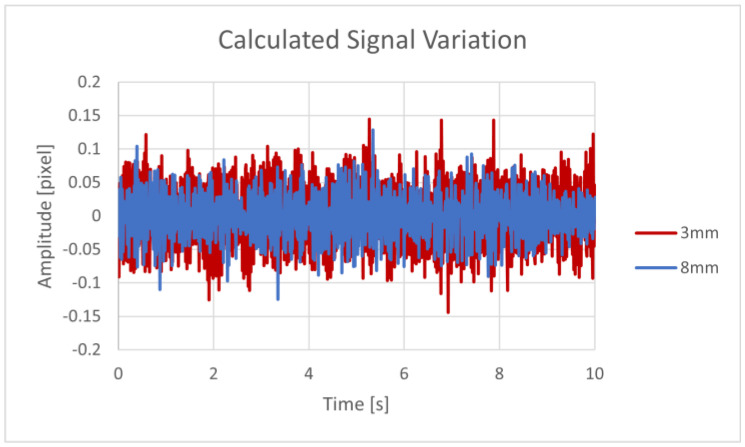
Signal amplitude variation over time for the 8 mm and 3 mm leak diameter scenarios.

While this method of data processing makes it easy to compare many tests, the raw signal data are more information-dense than the single-value average. The relation of the signal amplitude and leak flow from the recordings of the 400 µm covered fiber with leak diameters of 8 mm and 3 mm under a 1.05 L/min flow is presented in Figure 9.

The non-leaking system showed an inconsistent signal amplitude, often reaching the same level as that in the leaking system. This made it difficult to apply a statistical model to the data and accurately detect leaks. We tried to use amplitude thresholds to distinguish between normal and leaking operation, but the accuracy was barely better than that of random guessing. By analyzing and processing the data, we found that ML was more suitable for extracting information from the recordings. An additional factor potentially affecting leak flow determination could be the water temperature. To evaluate the impact of water temperature on the MMF output, we tested a 400 µm covered fiber under a leak flow of 5.7 L/min from a 6 mm hole at water temperatures of 7 °C, 18 °C, and 34 °C. We found that the average signal amplitude decreased by 25% between 7° and 34 °C and by 13% between 7 °C and 18 °C. While leak event discovery may not be affected by water temperature variation, for precise leak flow determination, the water’s temperature coefficient should be investigated.

### 4.5. Leak Detection with ML

To find an automated and reliable way to detect leaks, we applied ML methods. In the statistical analysis, the data were averaged to produce easy-to-analyze graphs; however, this process caused significant data loss. For ML data processing, we used the 1D signals from the previously analyzed speckle pattern videos. As each dataset was unique, the ML algorithm needed to be fine-tuned to produce the best predictions. The selected ML algorithms were KNN and SVM, with a fine setting for the KNN’s number of neighbors and a medium size for the SVM’s Gaussian function. Each recording was divided into 10 segments, and the number of training samples was multiplied. Overall, each test configuration included 30 samples containing 100 data points each. For the training, a common cross-validation technique was applied—K-fold (10-fold) cross-validation—to prevent the algorithms from overfitting. The model trained with K-1 subsets was validated using the remaining subset. This step was repeated ten times to ensure that each subset was validated exactly one time. We decided to use supervised learning for all of our ML testing. A total of 70% of the data were used to train the model, while 30% of the data were used to validate its accuracy. The duration of training and validation was about 10 s per algorithm.

**Data processing for the 400 µm MMF by ML**

**Figure 10:**
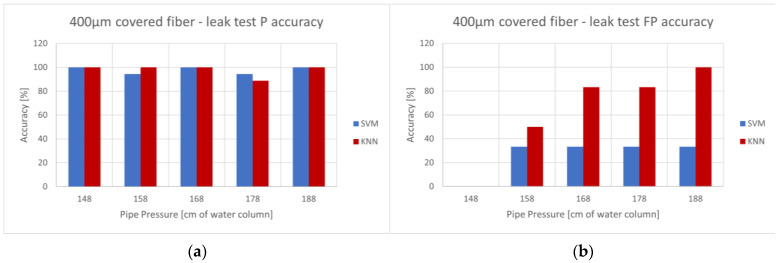
ML accuracy for leak detection—400 µm covered fiber. Positive accuracy shown in (**a**) and false positive rate shown in (**b**).

Preliminary data processing with ML showed that 400 µm MMF fiber is about twice as accurate as 200 and 600 µm fibers for leak size determination. The ML model training was performed separately for each pressure level, as the pipe pressure was not expected to fluctuate during typical use. While the data for the ML were separately classified according to leak size, we initially focused on the ability to detect a leak regardless of its size, which was the main goal of this research. The results for positive and false positive detection regardless of leak size for a given pressure—measured in cm of the water column—are shown in Figure 10 (P—percentage of leaks detected, FP—percentage of non-leaks classified as leaks).

While both algorithms detected the leaks with an accuracy above 85%, only the SVM algorithm delivered a pressure-consistent false positive rate under 35%. The KNN algorithm failed to differentiate regular flow from a leak at higher pressure levels; therefore, it is unreliable for the detection of leaks. The SVM algorithm’s accuracy was consistent for all measurements and fiber configurations, and it will be the focus of our further analyses.

**Figure 11:**
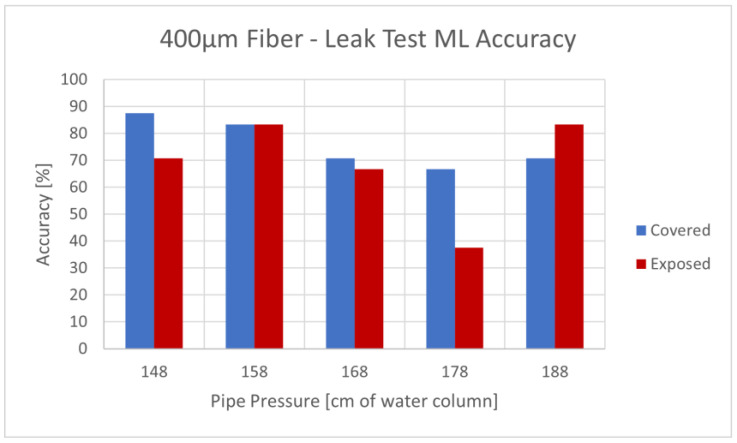
ML accuracy for leak size detection—400 µm covered and exposed fibers.

As mentioned, to train the ML models, we separated the data according to leak size, enabling the testing of leak size determination. The results for the leak size prediction accuracy for a given pipe pressure are shown in Figure 11.

The results were compared with random prediction as a threshold. With four classes tested (no leak and 3, 6, and 8 mm leak diameters), it was 25%. The accuracy was higher at lower pressures for the covered fiber, while for the exposed fiber, the accuracy was less consistent. Upon averaging the leak size determination accuracy of the SVM algorithm, we found an overall accuracy of 75.8% for the 400 µm covered fiber, 68.3% for the 400 µm exposed fiber, and 45.5% for the 200 µm covered fiber.

## 5. Discussion

This paper presents a novel technique for the continuous sensing of leakages in water pipes using the direct effect of the leaking water on the defocused speckle patterns recorded from covered and exposed fibers’ outlets.

We experimentally demonstrated that this type of sensor can detect outside intervention or shock to the pipe. By disturbing the pipe to simulate a pipe burst, the measured amplitude of the recorded signal spiked, exceeding the average signal amplitude during steady flow by 3 to 10 times. This result was consistent for the sensors fixed inside and outside the pipe for both covered and uncovered MMFs. Using a spectrogram, we exhibited that the frequency domain may also provide a method for the detection of pipe bursts and leaks.

Moreover, we were able to use the sensor data to detect leaks and determine their size with accuracy above 85% with the 400 µm MMF sensors, which were found to be optimal. Using both statistical methods and ML, we found that this sensor was sensitive enough to distinguish between leak sizes. Further improvement can be made by accounting for changes in water temperature and employing a more representative dataset for ML. Employing the selected ML algorithms allowed for the creation of an accurate model for detecting the existence of a leak. However, further investigation is required to eliminate false positive detection. We additionally demonstrated that defocusing of the digital camera when capturing the speckle patterns from the MMF outlet allowed us to increase the sensitivity and, therefore, leak detection capabilities of the sensor.

While showing that the developed fiber-based sensor is feasible, we acknowledge that improvements are required to expand and perfect its capabilities. A different method for calculating 1D variations in the speckle pattern recordings may include the measurement of the speckle pattern contrast. The ML detection accuracy may improve by using an extended dataset, lowering the false positive rate, and allowing leak detection regardless of pipe pressure. Another direction is the field of deep learning, where an algorithm can work with the original speckle pattern images. The ability of an uncovered fiber to collect information related to water turbidity and composition has yet to be explored. This will be especially useful in rural areas, where the water supply systems may suffer from pollution or contaminants. In addition to the detection of pipe bursts, the tested method also allows the detection of transient flow, water hammer, and pipe intervention and sabotage. Our method can be similarly useful for leak detection in oil and gas transmission systems. In addition, the fiber utilized for the sensor can be simultaneously used for communication by modulating the laser light.

A shortcoming of the current sensor is its lack of positional awareness. However, we aim to demonstrate distributed sensing by injecting photons from both ends of the fiber and generating a standing multi-modal wave. This allows us to change the observed location by tuning the position of the peaks of the standing wave with the relative frequency of the injected photons and scanning vibrations along the fiber in a continuous manner. Alternatively, the use of a Bragg filter (fiber-based grating) and a multi-wavelength laser could also determine leak location. This will enable the separation of specific wavelengths at different placements for measurement. The detection for each grating placement is possible using our method, as the interference between wavelengths is minimal in an MMF. By determining the particular wavelength affected by a pipe leak, its location can be evaluated.

## Figures and Tables

**Table 1:** MM optic fiber properties ^a^.

Fiber Properties	200 µm	400 µm	600 µm
Transmission region	400–2200 nm		
Core/Cladding material	Pure silica/Hard polymer		
Core/Cladding refraction index	1.458/1.365		
Core diameter	200 ± 5	400 ± 8	600 ± 10
Cladding diameter	225 ± 5	425 ± 8	630 ± 10
Coating diameter	500 ± 30	730 ± 30	1040 ± 30

^a^ From Thor Labs [34].

**Table 2:** Burst tests with the 400 µm inner fibers.

Test Number		No Leak–Maximum Amplitude [Pixel]	Simulated Burst–Maximum Amplitude [Pixel]	Relation
1	Covered fiber	0.122	0.567	4.67
	Exposed fiber	0.121	0.465	3.84
2	Covered fiber	0.116	0.321	2.77
	Exposed fiber	0.098	0.314	3.2

## Data Availability

The data presented in this study are available upon request from the corresponding author.
